# Cholesterol in the Cell Membrane—An Emerging Player in Atherogenesis

**DOI:** 10.3390/ijms23010533

**Published:** 2022-01-04

**Authors:** Karel Paukner, Ivana Králová Lesná, Rudolf Poledne

**Affiliations:** 1 Laboratory for Atherosclerosis Research, Centre for Experimental Medicine, Institute for Clinical and Experimental Medicine, 140 21 Prague, Czech Republic; ivka@ikem.cz (I.K.L.); rupo@ikem.cz (R.P.); 2 Department of Physiology, Faculty of Science, Charles University, 128 44 Prague, Czech Republic; 3 Faculty of Veterinary Medicine, University of Veterinary Sciences Brno, Small Animal Clinic, 612 00 Brno, Czech Republic; 4 Department of Anesthesia and Intensive Medicine, First Faculty of Medicine, Charles University and University Military Hospital, 128 08 Prague, Czech Republic

**Keywords:** cholesterol, macrophages, cell membrane

## Abstract

Membrane cholesterol is essential for cell membrane properties, just as serum cholesterol is important for the transport of molecules between organs. This review focuses on cholesterol transport between lipoproteins and lipid rafts on the surface of macrophages. Recent studies exploring this mechanism and recognition of the central dogma—the key role of macrophages in cardiovascular disease—have led to the notion that this transport mechanism plays a major role in the pathogenesis of atherosclerosis. The exact molecular mechanism of this transport remains unclear. Future research will improve our understanding of the molecular and cellular bases of lipid raft-associated cholesterol transport.

## 1. Introduction

A century of cholesterol research has revealed, step by step, the dietary cholesterol effect in experimental atherosclerosis [1], the relationship between atherosclerosis and dietary fat consumption [2], and cholesterol physiological synthesis [3]. Furthermore, low-density lipoprotein (LDL) has been identified as a carrier of cholesterol in the circulation [4], and high LDL levels as a key risk factor of atherosclerosis and myocardial infarction [5]. More recently, the importance of the function of a specific LDL receptor in the regulation of blood cholesterol levels has been described (reviewed in [6]). Lately, the increased use of statins to lower the levels of LDL-cholesterol [7] and the proprotein convertase subtilisin-kexin type 9 inhibitor (iPCSK9) disruptor blockade approach have been described.

Cholesterol and the phospholipid components of the cell membrane are important for normal cell function, and erratic lipid distribution or metabolism can have serious consequences for cells and the body. The membrane has a number of most important functions, such as serving as a permeability barrier and signaling through phosphoinositides. Numerous authors have shown that the biophysical characteristics of the membrane bilayer can have major effects on the properties of membrane proteins [8]. Changes in lipid organization can largely affect various cellular functions, such as membrane trafficking or signal transduction. These membrane-related effects can cause disease in living organisms due to genetic alterations, environmental factors (e.g., high dietary intake of saturated fats), or both [9]. The membrane is essential for cell existence, with cholesterol being an important determinant of membrane organization. Cholesterol is obtained mostly from food, and synthesized by almost all cells [10], but predominantly the liver and intestine. The liver also plays a crucial role in the availability of lipoproteins, the main cholesterol acceptors and carriers.

Its homeostasis is maintained through various mechanisms. The recommended total serum cholesterol level is under 5 mmol/L. Hypercholesterolemia has been successfully managed by statins over decades. However, recent data also suggest that the anti-inflammatory effect of statins directly impacts the macrophage phenotype, and the proportion of macrophage subpopulations in adipose tissue. The rates of cholesterol influx versus efflux through the cell membrane can affect the cholesterol content of lipid rafts in the cell membrane and may influence macrophage polarization and their inflammatory status. The role of the cell membrane in reverse cholesterol transport (RCT) (see below) is yet to be clarified. Cholesterol dysfunctionality may lead to structural and functional modifications, resulting in proatherogenic conditions and plaque progression up to the development of overt atherosclerosis. In this review, we will briefly summarize cholesterol trafficking and its influence on membrane organization and macrophage functions.

## 2. Cholesterol Homeostasis

The mammals have developed sophisticated and complex mechanisms to maintain plasma cholesterol levels, as well as cell membrane cholesterol levels, within a narrow physiological range [11]. In humans, one of the most significant factors affecting cell membrane functions and structure is the dietary intake of various fatty acids and cholesterol, which are subsequently delivered to cells throughout the body by lipoproteins. Cholesterol levels in the cell must be maintained within a homeostatic range; when these homeostatic mechanisms are weakened, such as in the later stages of atherosclerosis development, the consequences can be serious. Cholesterol imbalance leads to changes in membrane lipid organization, to symptoms of illness and, in the case of atherosclerosis, for example, to plaque progression [9]. 

Despite the important role of cholesterol in various cellular functions, regular plasma cholesterol levels in the populations of industrialized countries significantly exceed the physiological requirements. The influence of excess cholesterol intake on macrophage properties was demonstrated in an experimental model, where atherosclerosis-prone apolipoprotein E-deficient mice were fed the Western pattern diet. Within several days, circulating murine monocytes crossed the endothelium and increased their influx to the intima [12]. In humans, the complex interplay between the genotype and the phenotype was documented in a long-term analysis of the lifespan of a large Dutch family with a monogenic disease called familial hypercholesterolemia (FH). This study was possible thanks to the complete registry of all births and deaths, as well as marriages, since the mid-19th century. All three selected subjects had an identical disorder of the LDL receptor; they were heterozygous for the *V408M* mutation, and their family tree consisted of seven generations. Results revealed a variable lifespan over time from the mid-19th century until recent times. Quite surprisingly, the average lifespan of FH individuals was longer compared with that of the average Dutch population in the 19th century. A change came around 1920 when food availability and animal fat consumption increased, whereas the lifespan of FH individuals (with increased LDL cholesterol levels) shrunk. This was true until the 1960s when the awareness of the risk of cholesterol increased and, currently, there is no difference in the lifespan between FH individuals and controls. One can speculate that, in the 19th century, mortality from factors other than atherosclerosis-related causes, mainly infectious diseases, was so high that people died before they were able to develop cardiovascular disease. Hence, hypercholesterolemia and induced macrophage phagocytic activity may have conferred a survival advantage when infectious disease was prevalent [13,14,15]. 

Given the above, it may have been the effect of the diet high in cholesterol after the Second World War which caused the increase in mortality in FH individuals. The recurrence of excess mortality close to the mean age was probably related to treatment with statins [13]. This hypothesis was supported by experiments with genetically modified mice with high cholesterol levels, documenting increased protection against Gram-negative infections [16]. Together with the accelerated migration of immune cells into the arterial wall, elevated levels of LDL cholesterol particles and low levels of high-density lipoprotein (HDL) particles are believed to be among the major risk factors for the development of atherosclerosis [17]. Recently, the synergistic effect of monocyte properties and their cholesterol intracellular content was reported [18]. High-sensitivity C-reactive protein (hsCRP) is a common marker of inflammation. Data from Ridker’s studies suggest the key role of inflammation in the development of atherosclerosis and cardiovascular events. One of the studies demonstrated that the rate of death from ischemic heart disease (IHD) of individuals with low hsCRP levels (<1 mg/100 mL) was significantly lower, whereas those with high hsCRP levels (>3 mg/100 mL) showed a higher IHD prevalence [19,20]. This finding documented the synergy of high intravasal cholesterol levels and the pro-inflammatory status of the body. In fact, all patients with low hsCRP levels after statin therapy were reported to have more favorable clinical outcomes than those with higher hsCRP levels, despite significant reductions in LDL-cholesterol levels [21]. This “residual inflammatory risk” seems to be a suitable target for pharmacological treatment [22]. In addition, hsCRP levels provide significant prognostic information on one’s first cardiovascular event [19,20]. In a recent clinical study (CANTOS), treatment with interleukin 1β monoclonal antibodies (canakinumab) led to the effective reduction of not only hsCRP levels, but also, myocardial infarction-related mortality rates [23]. It is necessary to stress that although the recent use of hsCRP as an inflammation marker is important for epidemiological and clinical research, it is not informative in individual cases.

Not only do high atherogenic lipoprotein particles influence macrophage changes in both adipose tissue and the arterial wall [24], but the acute-phase response downregulates RCT [25,26]. It is now well established that the atherosclerotic plaque contains both pro-inflammatory (M1) and anti-inflammatory (M2) macrophages. The central role of macrophages in the pathophysiology of atherosclerosis has drawn attention to their properties in plaque initiation and progression, as well as regression. Data reported to date have demonstrated that the diet-induced disadvantage of elevated intravascular cholesterol levels acts in synergy with pro-inflammatory macrophage polarization by increasing cholesterol influx into the arterial wall [27].

## 3. Cholesterol Transport

Cholesterol accumulation within the cell is the result of an imbalance between the delivery to and removal of cholesterol from cells by lipoproteins. Delivery of cholesterol occurs by forward cholesterol transport, with apoB-containing lipoproteins, mainly native or modified LDL [28], playing a key role in the delivery of cholesterol esters to cells by receptor-mediated endocytosis. The removal of cholesterol from cells occurs via RCT, whereby the free cholesterol is transferred from cells to apolipoprotein A-1 (apoA-I), the major constituent protein in HDL. Cholesterol is subsequently esterified and exchanged within circulating lipoproteins, and transported to the liver and intestine for excretion. In this way, HDL particles (probably their small subfraction) play a pivotal role in the maintenance of cellular cholesterol homeostasis and, hence, in preventing the development of hypercholesterolemia and atherosclerosis. The fact that most of the cholesterol in our body is transported as cholesterol esters with plasma lipoproteins, serving as the main vehicle for cholesterol transport, are considered the two key elements of this current paradigm [29]. However, several recent studies have suggested that the transport of free cholesterol between its various pools within the body is much faster, and may be quantitatively more important than the transport of esterified cholesterol to be excreted via the liver [30] and intestine [31,32]. In addition, plasma lipoproteins are in constant direct contact with blood cells and endothelial cells of the vascular wall, which contain considerable amounts of free cholesterol, and may therefore significantly affect plasma cholesterol transport [29].

Recently, it was hypothesized that erythrocytes may also participate in RCT [33]. A study using cholesterol efflux capacity assays, the process that mediates the removal of excess cholesterol from foam cells to apoA-I or HDL, demonstrated that erythrocytes can exchange cholesterol with HDL in a bidirectional manner. However, only one-directional exchanges from erythrocytes towards apoA-I have been documented to date. In this study, specifically, erythrocytes, HDL and apoA-I, were obtained from human blood samples. Cell culture was prepared from the THP-1 monocyte line after loading with 3H-labeled cholesterol to imitate foam cells. These parts of the experiment together suggested that erythrocytes may play a significant role in RCT-mediated cholesterol efflux, as a temporary cholesterol store [34]. Another recent study [29] using erythrocytes indicated an active role of erythrocytes in RCT. In this study, erythrocytes were incubated with autologous plasma and the net movement of cholesterol from erythrocytes to plasma lipoproteins was demonstrated. A detailed analysis of cholesterol distribution among lipoprotein fractions revealed the net movement of cholesterol from erythrocytes to HDL and net movement of cholesterol from LDL to erythrocytes. In addition, in experiments with isolated lipoproteins, cholesterol movement was minimal after erythrocyte incubation with phosphate-buffered saline (PBS), very low-density lipoprotein (VLDL), or lipoprotein-depleted plasma [29]. The movement was unrelated to cholesterol esterification in the plasma: it was rapid, unaffected by the inhibition of lecithin–cholesterol acyltransferase (LCAT), with its plasma levels being the rate-limiting factor. The movement of cholesterol from erythrocytes to HDL and from LDL to erythrocytes suggested that this cholesterol flux could be part of reverse, rather than forward, cholesterol transport. It would also, therefore, seem unlikely that the cholesterol flux was the result of the detachment of cholesterol from the plasma membrane, or the secretion of exosomes from the erythrocytes. Thus, erythrocytes may take up free cholesterol from LDL and, presumably, from modified LDL as well, possibly lowering the amount of cholesterol which would be delivered to cells in the vessel wall. Erythrocytes may also transport cholesterol directly, by feeding the transport pathway to the intestine or liver for excretion, or conversion to bile acids. Erythrocytes are non-nucleated cells, and the half-life of most cholesterol transporters is much shorter than that of erythrocytes [29]. However, the dynamics and mechanism of cholesterol transport between erythrocytes and plasma lipoproteins have not been fully elucidated yet.

## 4. The Macrophage–Lipoprotein Interaction in Atherogenesis

The central role of monocytes and macrophages in atherogenesis was first described in the pioneering work of Goldstein and Brown, scientists who detailed the regulation of plasma cholesterol by specific apoB receptors [35]. They explained the function of scavenger receptors for apoB-containing particles on the surface of macrophages [36]. Macrophages with this receptor clean the subendothelial space of large and medium-sized arteries of abundant LDL particles. Macrophages, therefore, are able to maintain the physiological balance of the arterial wall between the current demand for cholesterol molecules on the one hand, and adequate or inadequate influx of LDL-carrying particles in hypercholesterolemia on the other [37]. In susceptible areas of medium-sized arteries that are prone to the permeation and subendothelial retention of LDL and remnant lipoproteins, atherosclerotic plaques may form [38]. Moreover, obese adipose tissue affects the adhesion of monocytes to endothelial cells, and their migration to the arterial wall. Adipokines produced in adipose tissue stimulate the production of adhesion molecules, and subsequently increase the adhesion of monocytes to the endothelium [39,40], followed by entry of monocytes into the subendothelial space [41,42]. Macrophage-trapping in the subendothelial space of the vessel wall is also influenced by proteins produced by the macrophages themselves (such as the macrophage colony-stimulating factor, MCSF and macrophage chemotactic factor, MCF).

The differentiation of monocytes into macrophages and, next, into resident macrophages in the subendothelial space of arteries is a most important process in atherogenesis. The development of macrophages into foam cells is always associated with LDL phagocytic activity so, understandably, the production of foam cells is greatly potentiated by abundant LDL particles within the arterial wall. Likewise, atherogenesis is enhanced by a decrease in RCT activity [43], and an increase in the intravascular levels of triglycerides and their accumulation in macrophages of the arterial wall, whereby remnants of very-low-density lipoprotein (VLDL) particles enter the arterial wall and become trapped in macrophages [44]. The recruitment of foamy monocytes to the inflamed endothelium-expressing vascular cell adhesion molecule-1 (VCAM-1) contributes to plaque formation during atherogenesis. Lipid uptake and CD11c activation are early and critical events in signaling the adhesive function of very late antigen-4 (VLA-4) on foamy monocytes, which can recruit monocytes through VCAM-1 on the inflamed arterial endothelium [12]. Lipoprotein inflow promotes the intracellular accumulation of lipid droplets in the cytoplasm to form foam cells, and the inflammatory cellular response. The process continues over a long period of time, up to many years, and may be amplified through the further promotion of lipoprotein retention, and the attraction of more white blood cells [38].

Differentiation of monocytes into residential macrophages is regulated by several transcription factors [45,46,47]. One of the significant differentiation factors is caveolin (a structural protein of intracellular caveolae), which stimulates the transcription of several pro-inflammatory mediators and surface receptors of macrophages [48]. Caveolin promotes the synthesis of CD36 (fatty acid translocase) on the macrophage surface [49,50]. This transmembrane protein mediates the caveolar endocytosis or phagocytosis of oxLDL in macrophages. Moreover, caveolin, CD36, and CD14 were cofractionated in the lipid raft [26,50,51,52]. In this way, CD36 could contribute to the retention of macrophages in atherosclerotic lesions and their subsequent conversion into foam cells [53,54]. One may also speculate on the role of the macrophage-producing protein netrin-1, a neuroimmune guidance cue, which is also abundantly expressed by macrophage foam cells in atherosclerotic plaques, where its expression promotes the accumulation of macrophages and disease progression [55]. The expression of netrin-1 is stimulated intracellularly by the effect of saturated fatty acids (SAFAs) such as palmitate [56]. This may indicate an atherogenic effect of SAFAs in addition to their well-known effect on hypercholesterolemia. In addition to the transcription factors, the non-coding RNAs represent yet another form of regulation of monocyte differentiation [57].

Another regulatory factor leading to the differentiation of monocytes into residential macrophages is the modification of circulating lipoproteins, with the modification of LDL cholesterol, namely desialylation (cleavage of sialic acid from native LDL), seemingly being the most fundamental [58]. Sialic acid represents the terminal carbohydrate of biantennary sugar chains in apolipoprotein B. In this step, LDL becomes smaller and denser than native LDL [59,60]. Desialylated LDL is more sensitive to oxidation [61] and self-association [62]. Modified LDL is more prone to be deposited in the vessel wall compared with the native LDL; these clusters are also more likely to be phagocytosed [63]. All of these events aid in the development of an inflammatory condition and, ultimately, the development of atherosclerosis.

## 5. Cholesterol in Cell Membrane and Rafts

**Figure 1:**
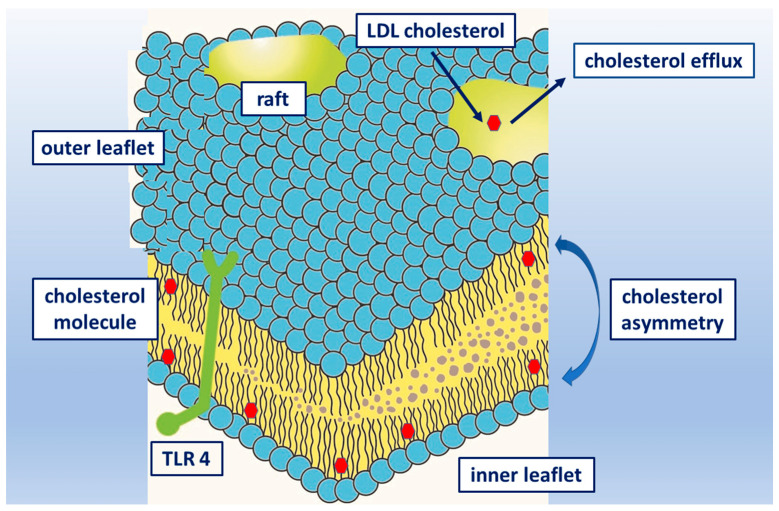
Schematic phospholipid bilayer cell membrane with cholesterol molecule (red symbol) and location of transmembrane receptor. Cholesterol asymmetry represents different proportions of cholesterol in the outer and inner phospholipid layers. Transport of cholesterol to the surface raft from plasma lipoproteins and possible reverse efflux.

As described above, cholesterol is a major lipid in the plasma membrane of mammalian cells, and plays diverse structural and functional roles. Cellular unesterified cholesterol is primarily (up to 90%) localized in the plasma membrane [64] and is essential for its physical integrity. Cholesterol has been implicated in the structural and functional modulation of integral membrane proteins and in the formation of cholesterol-rich membrane domains called membrane (lipid) rafts (Figure 1). It affects the physiological features of the cell membrane by controlling its fluidity, and regulating the negative membrane curvature through interaction with phospholipid acyl chains [65]. Another essential interaction is hydrogen bond formation between cholesterol and sphingomyelin. Lateral interactions of cholesterol with SAFAs or glycosylated lipid species divide the membrane into two distinct liquid phases. These interactions are essential for creating liquid-ordered membrane microdomains [66]. First, cholesterol in the inner leaflet of the plasma membrane may regulate various cell signaling pathways by specifically interacting with cytosolic scaffold proteins in a stimulus-dependent manner [67]. Second, the inner leaflet cholesterol levels were shown to modulate neurotransmitter receptor trafficking [68], the cytoskeleton and motility [68] and the intercellular behavior of cells [69]. Collectively, these studies point to a potential link between the inner leaflet cholesterol levels and diverse cellular processes, and suggest the importance of the transbilayer asymmetry of plasma membrane cholesterol. The asymmetry is maintained by the active transport of cholesterol from the inner leaflet to the outer one, and its chemical retention in the outer leaflet. Finally, the increase in cholesterol levels in the inner leaflet is triggered in a stimulus-specific manner, with cholesterol serving as a signaling lipid. The transbilayer asymmetry of plasma membrane cholesterol and the stimulus-induced plasma membrane cholesterol redistribution are crucial for tight regulation of the cellular processes under physiological conditions [70]. Despite recent data, the concept of the transbilayer distribution of cholesterol in the plasma membrane of mammalian cells is still considered controversial, partly because the assumed lipid rafts have not been visualized in the living cell yet. Analysis using radiometric cholesterol sensors have shown that the available cholesterol levels in the inner leaflet of the plasma membrane were low in unstimulated cells, and increased in a stimulus-specific manner to trigger cell signaling events. These differences support the hypothesis that cholesterol in the inner leaflet is kept low in unstimulated cells. The basal inner leaflet cholesterol levels vary significantly among cells [71]. The most recent data have demonstrated that the asymmetric distribution is maintained by ABCA1 (ATP-binding cassette transporter ABCA1) and ABCG1 (ATP-binding cassette sub-family G member 1) and their ATP-dependent cholesterol flopase activity, and by the ability of sphingomyelin in the outer leaflet to deter the reverse translocation of cholesterol to the inner leaflet [71].

Despite the above controversy, there is general agreement that unstable nanoscale assemblies within the membrane, primarily oligomeric protein–cholesterol–lipid complexes, associate into larger liquid-ordered functional nanodomains, also known as lipid (membrane) rafts. Lipid rafts are defined as small (10–200 nm) heterogeneous, highly dynamic cholesterol-, sphingolipid- and protein-enriched domains that compartmentalize cellular processes [72]. Lipid rafts are dynamic assemblies of both proteins and lipids, which contain various receptors and regulatory molecules, and act as a signal transduction platform. The rafts float freely within a liquid-disordered bilayer of cellular membranes and can cluster to form larger ordered domains. Several distinct classes of proteins are commonly incorporated—true resident proteins (glycosylphosphatidylinositol-linked proteins, caveolin, flotillin), signaling proteins (doubly acylated proteins like *Src* family kinases), G-protein-coupled receptor (GPCR) proteins, cholesterol linked and palmitoylated and myristoylated proteins [73]. Proteomic analyses of membrane lipid raft composition have shown that, in addition to the dominant presence of sphingolipids (mostly sphingomyelin), cholesterol, and glycolipids, and other phospholipids, including some species of phosphatidyl serine and phosphatidyl ethanolamine (PE), with mostly fully saturated or monounsaturated acyl chains, are also present [74,75].

Two types of lipid rafts are generally described: planar lipid rafts (also known as non-caveolar) and caveolae. Planar rafts form continuous non-invaginated membrane domains lacking any distinguishing morphological features, while caveolae are flask-shaped invaginated membrane structures formed by caveolin protein polymerization. Caveolins are transmembrane palmitoylated proteins forming a hairpin-like structure that binds tightly to cholesterol [76]. Three isoforms (caveolin-1, -2 and -3) transcribed from different genes have been identified. Of the three caveolins, caveolin-1 is known to modulate inflammatory responses [77]. Signal transduction is of particular importance in white blood cells, and upstream in the signaling pathway, many membrane receptors are functional only as complexes that assemble with specific lipid species [78].

The lipid rafts serve as a platform for signal transduction, particularly in the immune and inflammatory responses; for example, the T-cell antigen receptor (TCR) is a multisubunit immune recognition receptor, crucial for the adaptive immune response involved in signaling and engaging the lipid rafts in the process. T-cell antigen receptor activation changes the properties of the TCR complex and linked cytoplasmic raft-associated proteins, with the assembly becoming detergent-resistant upon TCR activation. Cholesterol depletion by methyl-beta-cyclodextrin dissociates these proteins from the lipid rafts, and inactivates the TCR signaling cascade. Moreover, the lipid rafts also function to concentrate major histocompatibility complex (MHC) class II molecules, loaded with specific peptides on the surface of antigen-presenting cells [73]. Given the lipid raft involvement in immune cell signaling, it is not surprising that lipid raft alterations are commonly found to be associated with the pathogenesis of various human diseases [79]. Knockdown of the caveoline-1 protein in an animal model was shown to lead to innate immunity defects, with affected mice becoming more susceptible to infection [77]. A study analyzing adipose tissue macrophages from living human kidney donors revealed the pro-inflammatory effect of palmitic and palmitoleic acids [80]. The proportion of pro-inflammatory CD14 + CD16 + CD36^high^ adipose tissue macrophages increases in visceral adipose tissue with the rising proportions of palmitic and palmitoleic acids in the phospholipids of the cell membrane [75]. Likewise, the proportion of these pro-inflammatory macrophages in adipose tissue increases with the major cardiovascular risk factors [81]. In our recent review, we suggested that pro-inflammatory macrophages participate in synergy with higher LDL-cholesterol levels in atherogenesis [82]. Toll-like receptors (TLRs) are part of the innate immune system that acts as the primary sensor of pathogens, and research has demonstrated that the integrity of lipid rafts is crucial for normal TLR signaling. Tsai et al. broadly showed that caveolin-1 regulates not only TL4 signaling, but also CD14, CD36 and MyD88 protein expression in macrophages and their response to bacterial infection [50]. Using cyclodextrin (for cholesterol depletion), Molfetta et al. reported that lipid raft integrity is essential for receptor ubiquitination and endocytosis, which is further involved in downstream signaling [83]. Cytokine signaling is altered by the disruption of cellular lipid rafts, attenuating the cytokine response. Decreased interferon gamma (INFγ) signaling was observed in *Leishmania donovani*-infected macrophages of Kala-azar patients. *L. donovani* increases membrane fluidity and perturbs the INFγ receptor (IFNGR1 and INFGR2) subunit assembly of the receptor occurring in normal macrophage lipid rafts. The depletion of macrophage membrane cholesterol by exogenous liposomal delivery restores INFγ signaling in infected macrophages [84].

Due to the lipid rafts’ increased cholesterol content, raft domains exhibit lower fluidity than the surrounding membrane [74]. The cell membranes of solid tumors, such as breast cancer, contain higher levels of cholesterol compared with normal cell membranes, with the implication being that larger raft domains can form in those membranes. This may stimulate signaling pathways to promote tumor growth and progression. An emerging area of research indicates a role of membrane rafts in neoplastic or tumor cell growth and invasiveness [74]. In accordance with this, an elevated level of blood cholesterol has been shown to increase the membrane raft content, promote tumor growth, and reduce apoptosis in prostate cancer [85]. Experiments with cholesterol depletion have largely improved our understanding of the effect of membrane raft disorganization on cellular activity. Since hypercholesterolemia and low-grade inflammation are key elements of atherogenesis, it is conceivable that the cholesterol (and its oxidized forms) content of lipid rafts likely influences the inflammatory signaling pathways, thereby modulating, among other things, the development of atherosclerosis. Indeed, the involvement of lipid rafts in several key steps in atherogenesis, such as the oxysterol-mediated apoptosis of vascular cells, attenuated the ability of HDL to exert anti-inflammatory effects and increased secretion of pro-inflammatory cytokines by white blood cells, has been documented [86].

## 6. Conclusions

In conclusion, is there any robust evidence that an increase in raft cholesterol of monocytes and macrophages is a significant player in general immune signaling? Although the relationship of a drop in LDL-cholesterol to pro-inflammatory parameters in a large clinical study was demonstrated more than a decade ago [87], its mechanism is still unclear. We already know that there is an intensive cholesterol molecule exchange on the cell surface [29,33,34] and that the plasma cholesterol levels increase the proportion of pro-inflammatory macrophages in human adipose tissue. On the other hand, stimulation of RCT by an HDL subfraction seems to have an opposite effect [88,89]. Unfortunately, any evidence of the presence of lipid rafts on the surface of a living cell is yet to be found. Further research is warranted to evaluate the mechanism and identify the role played by lipid raft cholesterol in atherogenesis.

## Abbreviations

apoA-I	apolipoprotein A-1
apoB	apolipoprotein B
FH	familial hypercholesterolemia
HDL	high-density lipoprotein
hsCRP	high-sensitivity C-reactive protein
IHD	ischemic heart disease
LDL	low-density lipoprotein
MHC	major histocompatibility complex
oxLDL	oxidized low-density lipoprotein
RCT	reverse cholesterol transport
SAFAs	saturated fatty acids
TLRs	toll-like receptors
VLDL	very-low-density lipoprotein

## References

[1] Anitschkow N.N., Chalatow S. (1913). Über experimentelle Cholesterinsteatose und ihre Bedeutung für die Entstehung einiger pathologischer Prozesse. Zent. Fur. Allg. Pathol. Und Pathol. Anat..

[2] Keys A., Anderson J.T., Grande F. (2016). Effect on Serum Cholesterol in Man of Mono-Ene Fatty Acid (Oleic Acid) in the Diet. Soc. Exp. Biol. Med..

[3] Bloch K. (1950). The Intermediary Metabolism of Cholesterol. Circulation.

[4] Gofman J.W., Glazier F., Tamplin A., Strisower B., Lalla O.D. (1954). Lipoproteins, Coronary Heart Disease, and Atherosclerosis. Physiol. Rev..

[5] Herrick J.B. (1944). An intimate account of my early experience with coronary thrombosis. Am. Heart J..

[6] Goldstein J.L., Brown M.S. (2015). A Century of Cholesterol and Coronaries: From Plaques to Genes to Statins. Cell.

[7] Scandinavian Simvastatin Survival Study Group (1994). Randomised trial of cholesterol lowering in 4444 patients with coronary heart disease: The Scandinavian Simvastatin Survival Study (4S). Lancet.

[8] Lee A.G. (2004). How lipids affect the activities of integral membrane proteins. Biochim. Biophys. Acta-Biomembr..

[9] Maxfield F.R., Tabas I. (2005). Role of cholesterol and lipid organization in disease. Nature.

[10] Cardoso D., Perucha E. (2021). Cholesterol metabolism: A new molecular switch to control inflammation. Clin. Sci..

[11] Goldstein J.L., Brown M.S. (2001). Molecular medicine. The cholesterol quartet. Science.

[12] Foster G.A., Xu L., Chidambaram A.A., Soderberg S.R., Armstrong E.J., Wu H., Simon S.I. (2015). CD11c/CD18 signals very late antigen-4 activation to initiate foamy monocyte recruitment during the onset of hypercholesterolemia. J. Immunol..

[13] Sijbrands E.J.G., Westendorp R.G.J., Defesche J.C., Meier P.H.E.M.d., Smelt A.H.M., Kastelein J.J.P. (2001). Mortality over two centuries in large pedigree with familial hypercholesterolaemia: Family tree mortality study. BMJ Br. Med. J..

[14] Poledne R., Zicha J. (2018). Human Genome Evolution and Development of Cardiovascular Risk Factors Through Natural Selection. Physiol. Res..

[15] Tall A.R., Yvan-Charvet L. (2015). Cholesterol, inflammation and innate immunity. Nat. Rev. Immunol..

[16] Netea M.G., Demacker P.N., Kullberg B.J., Boerman O.C., Verschueren I., Stalenhoef A.F., Meer J.W.V.d. (1996). Low-density lipoprotein receptor-deficient mice are protected against lethal endotoxemia and severe gram-negative infections. J. Clin. Investig..

[17] Libby P., Ridker P.M., Hansson G.K. (2011). Progress and challenges in translating the biology of atherosclerosis. Nature.

[18] Medbury H.J., Williams H., Li S., Fletcher J.P. (2015). The Bidirectional Relationship between Cholesterol and Macrophage Polarization. J. Clin. Cell. Immunol..

[19] Ridker P.M., Rifai N., Rose L., Buring J.E., Cook N.R. (2009). Comparison of C-Reactive Protein and Low-Density Lipoprotein Cholesterol Levels in the Prediction of First Cardiovascular Events. N. Engl. J. Med..

[20] Ridker P.M., Cook N. (2004). Clinical Usefulness of Very High and Very Low Levels of C-Reactive Protein Across the Full Range of Framingham Risk Scores. Circulation.

[21] Ridker P.M., Cannon C.P., Morrow D., Rifai N., Rose L.M., McCabe C.H., Pfeffer M.A., Braunwald E. (2009). C-Reactive Protein Levels and Outcomes after Statin Therapy. N. Engl. J. Med..

[22] Aday A.W., Ridker P.M. (2019). Targeting Residual Inflammatory Risk: A Shifting Paradigm for Atherosclerotic Disease. Front. Cardiovasc. Med..

[23] Ridker P.M., Everett B.M., Thuren T., MacFadyen J.G., Chang W.H., Ballantyne C., Fonseca F., Nicolau J., Koenig W., Anker S.D. (2017). Antiinflammatory Therapy with Canakinumab for Atherosclerotic Disease. N. Engl. J. Med..

[24] Ohtani Y., Irie T., Uekama K., Fukunaga K., Pitha J. (1989). Differential effects of alpha-, beta- and gamma-cyclodextrins on human erythrocytes. Eur. J. Biochem..

[25] Riottot M., Olivier P., Huet A., Caboche J.-J., Parquet M., Khallou J., Lutton C. (1993). Hypolipidemic effects of β-cyclodextrin in the hamster and in the genetically hypercholesterolemic rico rat. Lipids.

[26] Qin L., Zhu N., Ao B.X., Liu C., Shi Y.N., Du K., Chen J.X., Zheng X.L., Liao D.F. (2016). Caveolae and Caveolin-1 Integrate Reverse Cholesterol Transport and Inflammation in Atherosclerosis. Int. J. Mol. Sci..

[27] Stöger J.L., Gijbels M.J.J., Velden S., Manca M., Loos C.M., Biessen E.A.L., Daemen M.J.A.P., Lutgens E., Winther M.P.J.d. (2012). Distribution of macrophage polarization markers in human atherosclerosis. Atherosclerosis.

[28] Summerhill V.I., Grechko A.V., Yet S.F., Sobenin I.A., Orekhov A.N. (2019). The Atherogenic Role of Circulating Modified Lipids in Atherosclerosis. Int. J. Mol. Sci..

[29] Ohkawa R., Low H., Mukhamedova N., Fu Y., Lai S.-J., Sasaoka M., Hara A., Yamazaki A., Kameda T., Horiuchi Y. (2020). Cholesterol transport between red blood cells and lipoproteins contributes to cholesterol metabolism in blood. J. Lipid Res..

[30] Turner S., Voogt J., Davidson M., Glass A., Killion S., Decaris J., Mohammed H., Minehira K., Boban D., Murphy E. (2012). Measurement of Reverse Cholesterol Transport Pathways in Humans: In Vivo Rates of Free Cholesterol Efflux, Esterification, and Excretion. J. Am. Heart Assoc..

[31] Jakulj L., Dijk T.H., Boer J.F., Kootte R.S., Schonewille M., Paalvast Y., Boer T., Bloks V.W., Boverhof R., Nieuwdorp M. (2016). Transintestinal Cholesterol Transport Is Active in Mice and Humans and Controls Ezetimibe-Induced Fecal Neutral Sterol Excretion. Cell Metab..

[32] Boer J.F., Schonewille M., Dikkers A., Koehorst M., Havinga R., Kuipers F., Tietge U.J.F., Groen A.K. (2017). Transintestinal and Biliary Cholesterol Secretion Both Contribute to Macrophage Reverse Cholesterol Transport in Rats—Brief Report. Arterioscler. Thromb. Vasc. Biol..

[33] Hung K.T., Berisha S.Z., Ritchey B.M., Santore J., Smith J.D. (2012). Red Blood Cells Play a Role in Reverse Cholesterol Transport. Arterioscler. Thromb. Vasc. Biol..

[34] Lai S.-J., Ohkawa R., Horiuchi Y., Kubota T., Tozuka M. (2019). Red blood cells participate in reverse cholesterol transport by mediating cholesterol efflux of high-density lipoprotein and apolipoprotein A-I from THP-1 macrophages. Biol. Chem..

[35] Goldstein J.L., Brown M.S. (1987). Regulation of low-density lipoprotein receptors: Implications for pathogenesis and therapy of hypercholesterolemia and atherosclerosis. Circulation.

[36] Brown M.S., Goldstein J.L. (1983). Lipoprotein receptors in the liver. Control signals for plasma cholesterol traffic. J. Clin. Investig..

[37] Brown M.S., Goldstein J.L. (2003). Lipoprotein metabolism in the macrophage: Implications for Cholesterol Deposition in Atherosclerosis. Annu. Rev. Biochem..

[38] Tabas I. (2010). Macrophage death and defective inflammation resolution in atherosclerosis. Nat. Rev. Immunol..

[39] Cejkova S., Kubatova H., Thieme F., Janousek L., Fronek J., Poledne R., Kralova Lesna I. (2019). The effect of cytokines produced by human adipose tissue on monocyte adhesion to the endothelium. Cell Adhes. Migr..

[40] Bernardi S., Marcuzzi A., Piscianz E., Tommasini A., Fabris B. (2018). The Complex Interplay between Lipids, Immune System and Interleukins in Cardio-Metabolic Diseases. Int. J. Mol. Sci..

[41] Poledne R., Králová Lesná I., Čejková S. (2015). Adipose Tissue and Atherosclerosis. Physiol. Res..

[42] Postea O., Vasina E.M., Cauwenberghs S., Projahn D., Liehn E.A., Lievens D., Theelen W., Kramp B.K., Butoi E.D., Soehnlein O. (2012). Contribution of Platelet CX3CR1 to Platelet–Monocyte Complex Formation and Vascular Recruitment During Hyperlipidemia. Arterioscler. Thromb. Vasc. Biol..

[43] Reiss A.B., Cronstein B.N. (2012). Regulation of Foam Cells by Adenosine. Arterioscler. Thromb. Vasc. Biol..

[44] Bojic L.A., Sawyez C.G., Telford D.E., Edwards J.Y., Hegele R.A., Huff M.W. (2012). Activation of Peroxisome Proliferator-Activated Receptor δ Inhibits Human Macrophage Foam Cell Formation and the Inflammatory Response Induced by Very Low-Density Lipoprotein. Arterioscler. Thromb. Vasc. Biol..

[45] Schulz C., Massberg S. (2014). Atherosclerosis—Multiple Pathways to Lesional Macrophages. Sci. Transl. Med..

[46] Valledor A.F., Borràs F.E., Cullell-Young M., Celada A. (1998). Transcription factors that regulate monocyte/macrophage differentiation. J. Leukoc. Biol..

[47] Orekhov A.N., Sukhorukov V.N., Nikiforov N.G., Kubekina M.V., Sobenin I.A., Foxx K.K., Pintus S., Stegmaier P., Stelmashenko D., Kel A. (2020). Signaling Pathways Potentially Responsible for Foam Cell Formation: Cholesterol Accumulation or Inflammatory Response-What is First?. Int. J. Mol. Sci..

[48] Rudick M., Anderson R.G.W. (2002). Multiple Functions of Caveolin-1. J. Biol. Chem..

[49] Endemannl G., Stanton L.W., Madden K.S., Bryant C.M., White R.T., Protter A.A. (1993). THE JOURNAL OF BIOLOGICAL CHEMISTRV CD36 Is a Receptor for Oxidized Low Density Lipoprotein. J. Biol. Chem..

[50] Tsai T.H., Chen S.F., Huang T.Y., Tzeng C.F., Chiang A.S., Kou Y.R., Lee T.S., Shyue S.K. (2011). Impaired Cd14 and Cd36 expression, bacterial clearance, and Toll-like receptor 4-Myd88 signaling in caveolin-1-deleted macrophages and mice. Shock.

[51] Heit B., Kim H., Cosío G., Castaño D., Collins R., Lowell C.A., Kain K.C., Trimble W.S., Grinstein S. (2013). Multimolecular signaling complexes enable Syk-mediated signaling of CD36 internalization. Dev. Cell.

[52] Ring A., Le Lay S., Pohl J., Verkade P., Stremmel W. (2006). Caveolin-1 is required for fatty acid translocase (FAT/CD36) localization and function at the plasma membrane of mouse embryonic fibroblasts. Biochim. Biophys. Acta (BBA)-Mol. Cell Biol. Lipids.

[53] Febbraio M., Podrez E.A., Smith J.D., Hajjar D.P., Hazen S.L., Hoff H.F., Sharma K., Silverstein R.L. (2000). Targeted disruption of the class B scavenger receptor CD36 protects against atherosclerotic lesion development in mice. J. Clin. Investig..

[54] Thomas-Ecker S., Lindecke A., Hatzmann W., Kaltschmidt C., Zänker K.S., Dittmar T. (2007). Alteration in the gene expression pattern of primary monocytes after adhesion to endothelial cells. Proc. Natl. Acad. Sci. USA.

[55] Gils J.M., Derby M.C., Fernandes L.R., Ramkhelawon B., Ray T.D., Rayner K.J., Parathath S., Distel E., Feig J.L., Alvarez-Leite J.I. (2012). The neuroimmune guidance cue netrin-1 promotes atherosclerosis by inhibiting macrophage emigration from plaques. Nat. Immunol..

[56] Ramkhelawon B., Hennessy E.J., Menager M., Ray T.D., Sheedy F.J., Hutchison S., Wanschel A., Oldebeken S., Geoffrion M., Spiro W. (2014). Netrin-1 promotes adipose tissue macrophage retention and insulin resistance in obesity. Nat. Med..

[57] Javadifar A., Rastgoo S., Banach M., Jamialahmadi T., Johnston T.P., Sahebkar A. (2021). Foam Cells as Therapeutic Targets in Atherosclerosis with a Focus on the Regulatory Roles of Non-Coding RNAs. Int. J. Mol. Sci..

[58] Orekhov A.N., Tertov V.V., Mukhin D.N. (1991). Desialylated low density lipoprotein--naturally occurring modified lipoprotein with atherogenic potency. Atherosclerosis.

[59] Tertov V.V., Sobenin I.A., Orekhov A.N. (1992). Characterization of desialylated low-density lipoproteins which cause intracellular lipid accumulation. Int. J. Tissue React..

[60] Tertov V.V., Sobenin I.A., Gabbasov Z.A., Popov E.G., Jaakkola O., Solakivi T., Nikkari T., Smirnov V.N., Orekhov A.N. (1992). Multiple-modified desialylated low density lipoproteins that cause intracellular lipid accumulation. Isolation, fractionation and characterization. Lab. Investig..

[61] Tertov V.V., Kaplun V.V., Sobenin I.A., Orekhov A.N. (1998). Low-density lipoprotein modification occurring in human plasma possible mechanism of in vivo lipoprotein desialylation as a primary step of atherogenic modification. Atherosclerosis.

[62] Sobenin I.A., Tertov V.V., Orekhov A.N., Smirnov V.N. (1991). Synergetic effect of desialylated and glycated low density lipoproteins on cholesterol accumulation in cultured smooth muscle intimal cells. Atherosclerosis.

[63] Mezentsev A., Bezsonov E., Kashirskikh D., Baig M.S., Eid A.H., Orekhov A. (2021). Proatherogenic Sialidases and Desialylated Lipoproteins: 35 Years of Research and Current State from Bench to Bedside. Biomedicines.

[64] Lange Y., Swaisgood M.H., Ramos B.V., Steck T.L. (1989). Plasma membranes contain half the phospholipid and 90% of the cholesterol and sphingomyelin in cultured human fibroblasts. J. Biol. Chem..

[65] Yang S.-T., Kreutzberger A.J.B., Lee J., Kiessling V., Tamm L.K. (2016). The Role of Cholesterol in Membrane Fusion. Chem. Phys. Lipids.

[66] Sezgin E., Levental I., Mayor S., Eggeling C. (2017). The mystery of membrane organization: Composition, regulation and physiological relevance of lipid rafts. Nat. Rev. Mol. Cell Biol..

[67] Brachet A., Norwood S., Brouwers J.F., Palomer E., Helms J.B., Dotti C.G., Esteban J.A. (2015). LTP-triggered cholesterol redistribution activates Cdc42 and drives AMPA receptor synaptic delivery. J. Cell Biol..

[68] Pagler T.A., Wang M., Mondal M., Murphy A.J., Westerterp M., Moore K.J., Maxfield F.R., Tall A.R. (2011). Deletion of ABCA1 and ABCG1 Impairs Macrophage Migration Because of Increased Rac1 Signaling. Circ. Res..

[69] Frechin M., Stoeger T., Daetwyler S., Gehin C., Battich N., Damm E.M., Stergiou L., Riezman H., Pelkmans L. (2015). Cell-intrinsic adaptation of lipid composition to local crowding drives social behaviour. Nature.

[70] Liu S.-L., Sheng R., Jung J.H., Wang L., Stec E., O’Connor M.J., Song S., Bikkavilli R.K., Winn R.A., Lee D. (2017). Orthogonal lipid sensors identify transbilayer asymmetry of plasma membrane cholesterol. Nat. Chem. Biol..

[71] Buwaneka P., Ralko A., Liu S.-L., Cho W. (2021). Evaluation of the available cholesterol concentration in the inner leaflet of the plasma membrane of mammalian cells. J. Lipid Res..

[72] Pike L.J. (2006). Rafts defined: A report on the Keystone symposium on lipid rafts and cell function. J. Lipid Res..

[73] Simons K., Toomre D. (2000). Lipid rafts and signal transduction. Nat. Rev. Mol. Cell Biol..

[74] Hryniewicz-Jankowska A., Augoff K., Sikorski A.F. (2019). Highlight article: The role of cholesterol and cholesterol-driven membrane raft domains in prostate cancer. Exp. Biol. Med..

[75] Schuck S., Honsho M., Ekroos K., Shevchenko A., Simons K. (2003). Resistance of cell membranes to different detergents. Proc. Natl. Acad. Sci. USA.

[76] Parton R.G., Simons K. (2007). The multiple faces of caveolae. Nat. Rev. Mol. Cell. Biol..

[77] Medina F.A., Almeida C.J.d., Dew E., Li J., Bonuccelli G., Williams T.M., Cohen A.W., Pestell R.G., Frank P.G., Tanowitz H.B. (2006). Caveolin-1-Deficient Mice Show Defects in Innate Immunity and Inflammatory Immune Response during Salmonella enterica Serovar Typhimurium Infection. Infect. Immun..

[78] Cammarota E., Soriani C., Taub R., Morgan F., Sakai J., Veatch S.L., Bryant C.E., Cicuta P. (2020). Criticality of plasma membrane lipids reflects activation state of macrophage cells. J. R. Soc. Interface.

[79] Varshney P., Yadav V., Saini N. (2016). Lipid rafts in immune signalling: Current progress and future perspective. Immunology.

[80] Poledne R., Malinska H., Kubatova H., Fronek J., Thieme F., Kauerova S., Lesna I.K. (2019). Polarization of Macrophages in Human Adipose Tissue is Related to the Fatty Acid Spectrum in Membrane Phospholipids. Nutrients.

[81] Kralova Lesna I., Petras M., Cejkova S., Kralova A., Fronek J., Janousek L., Thieme F., Tyll T., Poledne R. (2018). Cardiovascular disease predictors and adipose tissue macrophage polarization: Is there a link?. Eur. J. Prev. Cardiol..

[82] Poledne R., Kralova Lesna I. (2021). Adipose tissue macrophages and atherogenesis—A synergy with cholesterolaemia. Physiol. Res..

[83] Molfetta R., Gasparrini F., Peruzzi G., Vian L., Piccoli M., Frati L., Santoni A., Paolini R. (2009). Lipid Raft-Dependent FcεRI Ubiquitination Regulates Receptor Endocytosis through the Action of Ubiquitin Binding Adaptors. PLoS ONE.

[84] Sen S., Roy K., Mukherjee S., Mukhopadhyay R., Roy S. (2011). Restoration of IFNγR Subunit Assembly, IFNγ Signaling and Parasite Clearance in Leishmania donovani Infected Macrophages: Role of Membrane Cholesterol. PLoS Pathog..

[85] Zhuang L., Kim J., Adam R.M., Solomon K.R., Freeman M.R. (2005). Cholesterol targeting alters lipid raft composition and cell survival in prostate cancer cells and xenografts. J. Clin. Investig..

[86] Lemaire-Ewing S., Lagrost L., Néel D. (2012). Lipid rafts: A signalling platform linking lipoprotein metabolism to atherogenesis. Atherosclerosis.

[87] Ridker P.M., Danielson E., Fonseca F.A., Genest J., Gotto A.M., Kastelein J.J., Koenig W., Libby P., Lorenzatti A.J., Macfadyen J.G. (2009). Reduction in C-reactive protein and LDL cholesterol and cardiovascular event rates after initiation of rosuvastatin: A prospective study of the JUPITER trial. Lancet.

[88] Wang S.h., Yuan S.g., Peng D.q., Zhao S.p. (2012). HDL and ApoA-I inhibit antigen presentation-mediated T cell activation by disrupting lipid rafts in antigen presenting cells. Atherosclerosis.

[89] Pirillo A., Bonacina F., Norata G.D., Catapano A.L. (2018). The Interplay of Lipids, Lipoproteins, and Immunity in Atherosclerosis. Curr. Atheroscler. Rep..

